# Early Results of Slanted Recession of the Lateral Rectus Muscle for Intermittent Exotropia with Convergence Insufficiency

**DOI:** 10.1155/2015/380467

**Published:** 2015-01-26

**Authors:** Bo Young Chun, Kyung Min Kang

**Affiliations:** Department of Ophthalmology, School of Medicine, Kyungpook National University, 50 Samduk-2 ga, Jung-gu, Daegu 700-721, Republic of Korea

## Abstract

The aim of this study was to evaluate the efficacy of slanted recession of the lateral rectus muscle for intermittent exotropia with convergence insufficiency. This prospective study included 31 patients who underwent slanted lateral rectus recession for intermittent exotropia with convergence insufficiency between June 2010 and June 2012. Following parameters were recorded and analyzed: patient sex, age, preoperative and postoperative near and distance ocular alignment, and changes in stereopsis. The mean age of the patients was 9.2 years. The preoperative mean deviation angle was 32.4 PD at distance and 43.4 PD at near. After 6 months, slanted lateral rectus recession reduced the deviation angles to 2 PD at distance and 3.4 PD at near. In addition, the mean difference between distance and near deviation angles was significantly reduced from 11 PD to 1.4 PD at 6 months postoperatively. Slanted lateral rectus recession for intermittent exotropia with convergence insufficiency in children successfully reduced the distance and near exodeviations and the near-distance difference without increasing the risk of long-term postoperative esotropia or diplopia.

## 1. Introduction

The convergence-insufficiency type of intermittent exotropia (IXT) is characterized by a greater exodeviation at near than at distance by at least 10 prism diopters (PD) [1, 2]. This type is uncommon in children with IXT, with a reported prevalence of 1.2 to 7.8% [3, 4]. The reported symptoms of IXT with convergence insufficiency include headache, diplopia, blurred vision, eye fatigue, and reading problems, as the condition is associated with weak fusional convergence amplitudes, a low accommodative convergence to accommodation ratio, tenacious distance fusional drive, and decreased accommodative amplitudes [1, 5]. Nonsurgical treatments such as orthoptic treatment, prisms, plus lens, and psychotherapy may relieve the symptoms [5, 6]. However, patients who do not respond to any combination of nonsurgical treatments or have too large exodeviation may require surgical treatment [7, 8].

The surgical procedures recommended for this type of IXT include bilateral lateral rectus (LR) recession with or without a slanting procedure, bilateral medial rectus (MR) resection with or without a slanting procedure, LR recession with MR resection, or MR resection with an adjustable suture [1, 5, 7–12]. Success rates of these procedures vary between 18 and 92%. In addition, some patients show postoperative distance overcorrection, near undercorrection, or limitation of abduction [10, 12].

Among the surgical procedures, slanted LR recession for the convergence-insufficiency type of IXT, which was introduced by Snir et al. [1], demonstrated the best success rate, at 92%. This surgical technique weakens the lower fibers of the LR more than the upper fibers; thus, the technique has a greater impact on the near exodeviation than on the distance exodeviation, thereby reducing the near-distance exodeviation difference [1]. This pilot study was conducted primarily using adults. No prospective study has evaluated the surgical results of slanted LR recession for children with IXT with convergence insufficiency. The aim of this study was to evaluate the efficacy of slanted recession of the LR muscle for IXT with convergence insufficiency in children.

## 2. Materials and Methods

This prospective institutional study consisted of patients with an IXT greater at near than at distance by 10 PD or more. Thirty-one patients who underwent bilateral slanted LR recession for IXT with convergence insufficiency between June 2010 and June 2012 were included. All patients had a follow-up duration greater than 6 months. The research was approved by the institutional review board of our institution before this study began and adhered to the Declaration of Helsinki for the protection of human subjects. Informed consent was obtained from the parents of all patients prior to surgery.

Patients with associated vertical, torsional, and A-V pattern of strabismus, previous ocular or strabismus surgery, ocular or neurologic pathologic conditions, amblyopia, or a postoperative follow-up duration less than 6 months were excluded from the study. Preoperatively, no patients underwent occlusion therapy or myopic overcorrection or received a prescription for prism eyeglasses.

All patients underwent full ophthalmologic and orthoptic evaluations, including best-corrected visual acuity and cycloplegic refraction (with cyclopentolate 1% instilled twice). The distance (6 m) and near (1/3 m) deviations were measured with the alternating prism cover test by using accommodative targets in all the nine diagnostic positions of gaze, while the patients were wearing their best optical correction. Abnormalities induction and version were checked. Torsion was measured with a double Maddox rod test and fundus photograph. A binocular test for fusion and stereopsis was performed for all patients. Fusion was evaluated by the Worth four-dot test at distance and near, and stereopsis was evaluated by the Titmus test at near.

**Figure 1:**
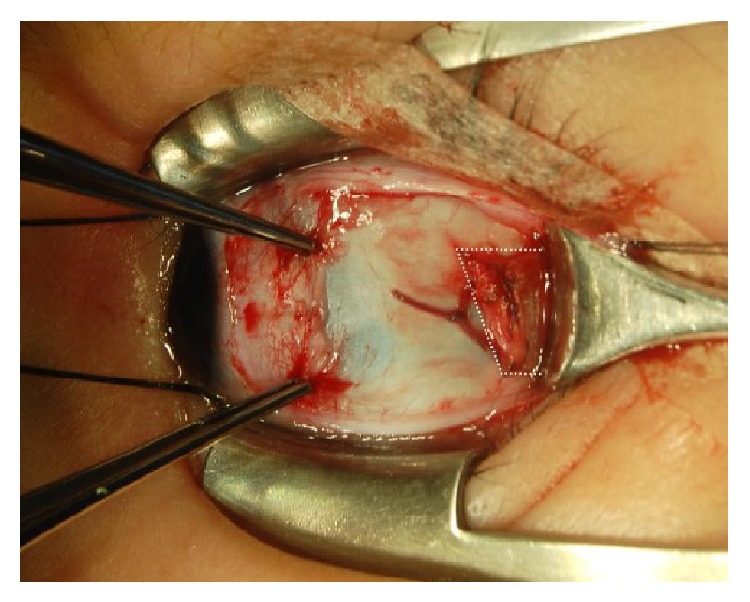
Slanted lateral rectus muscle recession. The upper pole was recessed according to the distance exodeviation and the lower pole was recessed according to the near exodeviation.

All patients were operated on by the same surgeon (BYC). Under general anesthesia, slanted LR recession was performed through a temporal limbal peritomy. In the slanted LR recession, the new insertion was created in an oblique fashion in comparison with the original insertion (Figure 1). The upper pole of the LR muscle was recessed according to the distance deviation angle (from 6.5 to 8.5 mm). The lower pole of the LR muscle was recessed according to the near deviation angle (from 8.0 to 9.5 mm); the lower pole was recessed more than the upper pole, as the deviation was greater at near than at distance (from 1.0 to 2.0 mm) [1]. The extent of the recession was primarily based on the length indicated by surgical tables derived from Parks.

All patients were examined at 1 day, 1 week, 1 month, 3 months, and 6 months postoperatively and at their last visit. The examination included the angles of deviation at distance and near, the extent of the difference between those two deviation angles, and checking whether there was postoperative diplopia, torsion, or A-V pattern of strabismus. The criteria for successful surgery were a postoperative deviation angle at near and distance of less than or equal to 8 PD and a difference between the near and distance deviation angles of less than or equal to 8 PD. The impact of slanted LR recession on the reduction of the near-distance difference was calculated as follows: the mean reduction of near-distance difference in exodeviation after slanted surgery was divided by the mean amount of slanted recession, which was defined as the mean difference between the new upper and lower poles of the slanted LR muscle compared with their original insertions [1].

Paired *t*-tests were used to compare the preoperative and postoperative angles of deviation at distance and near and the differences between them. Differences were considered significant if the *P* value was less than 0.05. The statistical analyses were performed using SPSS software (version 14.0, SPSS, Inc., Chicago, IL).

## 3. Results

In this prospective study, the study group was composed of 31 patients, including 14 males and 17 females, with a mean age of 9.2 years (range, 4~12 years). The mean duration of follow-up was 9.7 ± 5.5 months (range, 6~24 months) (Table 1).

The study group showed a significant postoperative reduction in the means of both the distance and near exodeviation. The preoperative mean exodeviation was 32.4 ± 4.8 PD (range, 25~40 PD) at distance and 43.4 ± 3.2 PD (range, 35~50 PD) at near. After 3 months, slanted LR recession reduced the mean exodeviation to 2.2 ± 3.8 PD (range, 0~14 PD) at distance and 3.1 ± 4.4 PD (range, 0~16 PD) at near, and, after 6 months, the procedure reduced the mean exodeviation to 2.0 ± 4.3 PD (range, −2~16 PD) at distance and 3.4 ± 5.0 PD (range, −2~18 PD) at near. At 6 months postoperatively, the number of patients with a distance deviation of less than or equal to 8 PD was 28 (90.3%), and the number of patients with a near deviation of less than or equal to 8 PD was 26 (83.9%).

In addition, the mean difference between the distance and near deviation angles was significantly reduced from 11.0 ± 2.0 PD (range, 10~15 PD) preoperatively to 1.4 ± 2.2 PD (range, 0~8 PD) at 6 months postoperatively (*P* < 0.001) (Table 2). Each millimeter of difference between the upper and lower poles of the slanted recessed LR muscle was associated with an improvement of 8.7 PD in the difference between the near and distance angles of exodeviation. At 6 months postoperatively, the number of patients with less than or equal to 8 PD difference between the distance and near deviations was 31 (100%).

A successful outcome was obtained in 26 of the 31 (83.9%) patients. The 5 patients who did not satisfy the criteria for a successful outcome demonstrated undercorrection of the exodeviation but still showed a postoperative reduction in the difference between the distance and near exodeviations of less than or equal to 8 PD. The mean stereopsis significantly increased from 496.7 arcsec preoperatively to 160 arcsec at 6 months postoperatively (*P* < 0.05). At the last follow-up, none of the patients demonstrated abduction limitation, diplopia, consecutive esotropia, torsion, or A-V pattern misalignment.

## 4. Discussion

In this study, we performed slanted recession of the bilateral LR muscles in IXT patients with convergence insufficiency. The residual near and distance exodeviations were less than or equal to 8 PD in 26 patients, and the mean near-distance exodeviation difference was reduced to 1.4 ± 2.2 PD at 6 months postoperatively. None of the patients demonstrated early postoperative overcorrection or diplopia, and there was no torsion effect or A-V phenomenon. Each millimeter of difference between the upper and lower poles of the slanted recession of the LR muscles improved the near-distance difference by 8.7 PD.

This excellent surgical outcome may be a result of the slanted LR recession, which was introduced by Snir et al. [1]. The authors suggested that the theoretical basis of the slanted recession of LR muscles was derived from Scott's investigations [1, 13]. Scott has reported that the fibers of the upper and lower poles of the LR muscles are of equal length in the primary ocular position [13]. However, in a down gaze position (30°) and at near vision, the upper muscle fibers are lengthened from 40.0 to 41.5 mm, and the lower muscle fibers are reduced from 40.0 to 37.1 mm [13]. Therefore, by recessing the lower muscle fibers more than the upper fibers, the exodeviation at near is decreased and the muscle tension of the upper and lower poles is balanced at the new insertion, which reduces the near-distance exodeviation difference [1, 13]. Snir et al. [1] reported that, after slanted recession of the LR in IXT patients with convergence insufficiency, the residual near and distance exodeviations were less than 8 PD in all patients but one, and the mean near-distance exodeviation difference was reduced to 2.9 ± 2.4 PD. The authors found that a 1 mm difference between the upper and lower poles of the slanted recession reduced the near-distance difference by 4.6 PD. They concluded that slanted LR recession was superior to standard recession at reducing both distance and near exodeviations and at reducing the near-distance difference.

Many authors have reported postoperative outcomes after surgical procedures for IXT patients with convergence insufficiency. However, most of the procedures demonstrated unsatisfactory success rates (range, 18~67%) or postoperative diplopia [10, 12, 14, 15].

Among six patients who underwent bilateral MR resections ranging from 4 to 6 mm, von Noorden [12] found that the near exodeviation was reduced to less than 10 PD in four patients. However, in five patients, Fresnel prisms were required to treat diplopia at distance for 5 weeks, and, in one patient, Fresnel prisms were required for 5 months. Haldi [14] performed the same technique ranging from 4 to 8 mm; the near exodeviation was reduced to less than 10 PD in 50% of the patients. Hermann [15] reported bilateral MR resections ranging from 3.5 to 4.5 mm in 13 patients; only 6 of the patients had a near deviation angle less than 10 PD, and 12 patients had esotropia at distance with diplopia that persisted for 9 or fewer months. Raab and Parks [10] performed bilateral LR recessions, but their success rate of near exodeviation was only 28% after 6 months.

To increase the surgical success rate for IXT with convergence insufficiency, Kraft et al. [2] performed the surgery in which the monocular strengthening of the MR resection exceeded the LR weakening; the amount of the MR resection was based on the near deviation; and the amount of the LR recession was based on the distance deviation. In all of the patients, the near deviation was corrected and the near-distance difference was reduced; however, 18% of the patients had consecutive esotropia, and all of the patients had limitation of abduction. Choi et al. [9] performed unilateral surgery, as described by Kraft et al. [2], and their overall success rate was 42.9%. The near-distance difference was reduced from 11.3 PD to 4.6 PD postoperatively. Akbari et al. [16] performed slanted MR resection to treat IXT with convergence insufficiency and reported that eleven of fifteen patients showed surgical success and that recurrent exotropia occurred in four patients. Biedner [17] performed a single MR resection in three patients with a 10 to 20 PD exodeviation at near and a 10 PD or higher exodeviation at distance. All of the patients showed postoperative alignment to within 10 PD in all fields of gaze.

A comparison of our postoperative results with previous reports yields several significant points. First, 26 patients (83.9%) demonstrated residual near and distance exodeviations with less than or equal to 8 PD after slanted LR recession. This success rate of slanted LR recession is relatively superior to the success rates that have been reported using other surgical procedures for IXT with convergence insufficiency, which are between 18% and 76.2% [2–4, 7–10, 12]. Second, the mean near-distance exodeviation difference was reduced from 11.0 PD to 1.4 PD, but still all of the patients demonstrated a near-distance exodeviation difference of less than or equal to 8 PD after the slanted LR recession. Our results correspond with the results of Snir et al. [1] who reported that slanted LR recession is superior to standard recession in reducing both distance and near exodeviations and in reducing the difference between near and distant exodeviations. Third, none of the patients demonstrated postoperative esotropia, diplopia, or abduction limitation, and there was no torsional effect or postoperative A-V phenomenon. The present study shows that, for correcting children's IXT with convergence insufficiency, slanted LR recession appears to be more effective in preventing postoperative amblyopia that is caused by postoperative overcorrection than other surgical procedures. To date, no study has examined the outcomes of slanted LR recession in IXT children with convergence insufficiency. At last, each millimeter of slanting between the upper and lower poles reduced the near-distance difference by 8.7 PD. These results are superior to those of Snir et al. [1] who reported that a 1 mm difference reduced the near difference by 4.6 PD. We only included children who had IXT with convergence insufficiency and who did not undergo periods of unsuccessful nonsurgical therapy. The difference in age criteria may be the cause of our superior postoperative results.

This study has important limitations, but most stem from its small sample size and the absence of a control group. In addition, children included in this study had relatively smaller near-distance difference of 11 PD than those of other studies (11.3 PD~14.5 PD) [1, 2, 7–9, 16]. We speculate that this relatively smaller preoperative near-distance difference may be one of the reasons for our superior surgical outcome compared to prior reports of surgeries in IXT with convergence insufficiency which were performed primarily in adults.

Interestingly, our results are different from those of van der Meulen-Schot et al. [18], who did slanting procedures in the opposite manner as done in this study and achieved good results. That slanting in opposite manners can give equal results suggests that slanting itself does not contribute. Therefore, Bothun [19] suggested that it was the recession of the extraocular muscles that was primarily responsible for the surgical effect, irrespective of muscle slanting. Kushner [20] also postulated that insertion slanting played no role in the results obtained in the published series of insertion slanting surgery. He presented it on the basis of sarcomere remodeling which suggests that, within weeks of an insertion slanting procedure, the edge tension difference should be equalized and the effect of the slanting is negated [20]. However, collapse of near-distance difference after slanted LR recessions was well maintained over postoperative 6 months in this study. The differential innervations of the superior and inferior halves of the LR might play a role in the effects of slanted recessions [21].

In conclusion, slanted LR recession for IXT with convergence insufficiency in children successfully reduced the distance and near exodeviations and the near-distance difference without increasing the risk of long-term postoperative esotropia or diplopia.

## Figures and Tables

**Table 1:** Preoperative patient characteristics.

Number of patients	31
Age (yrs) (mean ± SD; range)	9.2 ± 6.2 (4~12)
Gender, number (%)	
Male	14 (45%)
Female	17 (55%)
Follow-up period (months) (mean ± SD; range)	9.7 ± 5.5 (6~24)

**Table 2:** Change in the mean postoperative angles. The mean distance deviation, mean near deviation, and near-distance difference (mean ± SD, range) showed significant postoperative reductions (*P* < 0.01).

	Distance deviation (PD)	Near deviation (PD)	Near-distance difference (PD)
Before operation	32.4 ± 4.8 (25~40)	43.4 ± 3.2 (35~50)	11.0 ± 2.0 (10~15)
Postoperative 3 months	2.2 ± 3.8 (0~14)	3.1 ± 4.4 (0~16)	0.9 ± 1.6 (0~6)
Postoperative 6 months	2.0 ± 4.3 (−2~16)	3.4 ± 5.0 (−2~18)	1.4 ± 2.2 (0~8)

PD = prism diopters.
